# Advances and Challenges Regarding American Tegumentary Leishmaniasis in the Amazon: Scientific, Epidemiological and Public Health Perspectives

**DOI:** 10.1590/0037-8682-0564-2025

**Published:** 2026-07-31

**Authors:** Jorge Augusto de Oliveira Guerra, Débora Raysa Teixeira de Sousa, Silmara Navarro Pennini, Thamires Bastos Pinheiro, Melissa Melo Cavalcante, Luciana Mendes, Diego Carvalho, Fábio Borgonovo, Francisco Mateus João, Isolda Prado de Negreiros Nogueira Maduro, Luiz Carlos Cunha Marques, Silvia Cassia Brandão Justiniano, João Marcos Bemfica Barbosa Ferreira, Katia do Nascimento Couceiro, Maria das Graças Vale Barbosa Guerra

**Affiliations:** 1 Fundação de Medicina Tropical Doutor Heitor Vieira Dourado, Manaus, AM, Brasil.; 2 Universidade do Estado do Amazonas, Programa de Pós-graduação em Medicina Tropical, Manaus, AM, Brasil.; 3 Universidade do Estado do Amazonas, Programa de Ciências da Saúde da Amazônia, Manaus, AM, Brasil.; 4 Universidade do Estado do Amazonas, Programa de Pós-graduação em Ciências Aplicadas à Dermatologia, Manaus, AM, Brasil.; 5 Centro Universitário CEUNI-FAMETRO, Manaus, AM, Brasil.; 6 Universidade Federal do Amazonas, Manaus, AM, Brasil.; 7 Department of Infectious Diseases, Luigi Ospedale Luigi Sacco, University of Milan, Milan, Italy.

**Keywords:** Cutaneous leishmaniasis, Mucosal leishmaniasis, Epidemiology, Neglected disease, Amazon, Health planning

## Abstract

American tegumentary leishmaniasis (ATL) remains a significant public health problem in the Americas, with the Amazon region standing out in Brazil for its high number of cases. This article aimed to present advances in the understanding of epidemiology, diagnosis, treatment and control of ATL in the state of Amazonas over the past decades. This study is a narrative review of scientific studies published in the literature between 2011 and 2025, retrieved from databases such as PubMed and SciELO, as well as public data from the Notifiable Diseases Information System (SINAN), official reports from the Ministry of Health and technical notes from the Amazonas Health Surveillance Foundation. The evolution of disease incidence is reported, highlighting the predominance of *Leishmania (Viannia) guyanensis* and the environmental factors associated with the increase in cases, such as deforestation and unplanned urbanization. Advances in diagnostic strategies include the expanded use of molecular techniques and the strengthening of the primary healthcare network. Regarding therapy, clinical trials with drugs such as pentamidine, miltefosine, tamoxifen and itraconazole have demonstrated their efficacy in treatment. The review also includes socio-environmental, genetic and experimental studies that have contributed to a better understanding of the disease. It emphasizes the need to establish a priority agenda that includes the use of telemedicine and the institutionalization of collaborative research for therapeutic innovation, integration between research and healthcare services and strategies adapted to the Amazonian context. Regional scientific advances can support more effective control policies and reduce the burden of ATL in this highly endemic setting.

## INTRODUCTION

**FIGURE 1:**
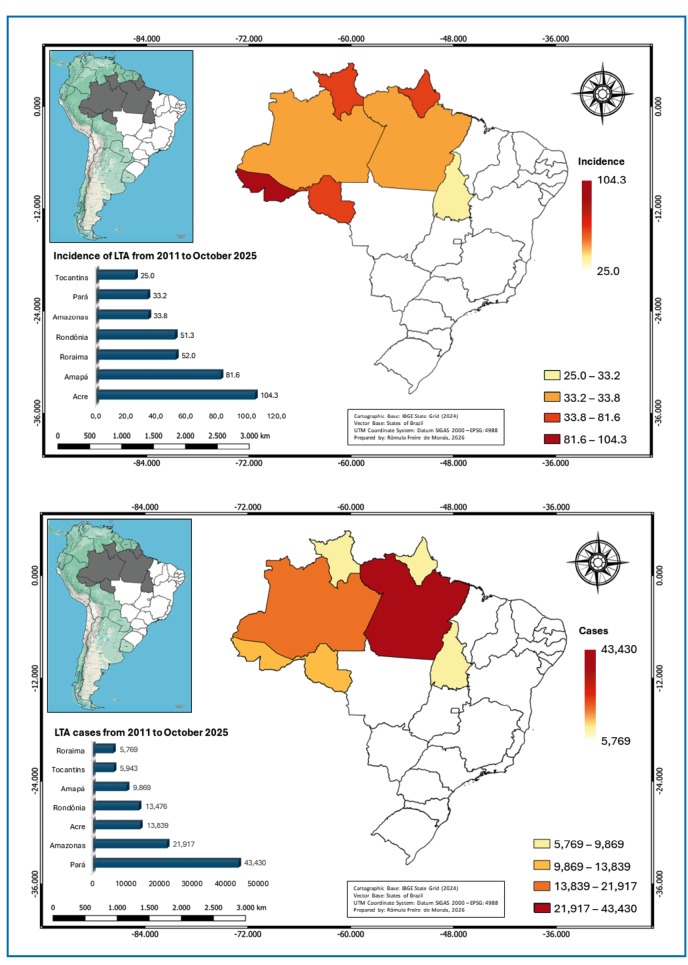
Distribution of the frequency (map on the left) and incidence (map on the right) of ATL cases in the Northern region of Brazil between 2011 and October 2025. Source: National System of Notifiable Diseases Information (SINAN), 2011 - October 2025.

American tegumentary leishmaniasis (ATL) is a neglected, non-contagious infectious-parasitic disease caused by approximately 20 species of protozoa of the genus *Leishmania* and is transmitted by phlebotomine sand flies. Each *Leishmania* species exhibits specific characteristics related to clinical manifestations, vectors, reservoirs, epidemiological patterns, geographical distribution and therapeutic response^1^ ^,^ ^2^. In Brazil, seven *Leishmania* species and more than two hundred species of phlebotomine sand flies are involved in transmission^3^ ^,^ ^4^, with foci associated with deforested areas, urban expansion, mining, oil production, and road construction. *L. (V.) braziliensis*, *L. (V.) guyanensis* and *L. (L.) amazonensis* are the most important species^5^. Although it has low lethality, leishmaniasis occurs predominantly in cutaneous and mucosal forms^4^. Endemic in 22 countries in the Americas, the disease has been reported from the southern United States to northern Argentina, with the exception of Chile and Uruguay, with 19 countries endemic for American tegumentary leishmaniasis (ATL) and 13 countries for visceral leishmaniasis (VL^1^ ^,^ ^6^ ^.^ In Brazil, ATL is reported in all states, and over the past ten years, a declining trend in case numbers has been observed. For the period between 2011 and 2025, the northern region recorded the highest number of cases, 117.729 (45,3%), an estimated mean annual incidence of 41.7 cases per 100,000 inhabitants^4^ (Figure 1). 

## METHODS

This study presents a narrative review of scientific studies published in the literature between 2011 and 2025 on American tegumentary leishmaniasis (ATL) in the state of Amazonas, retrieved from databases such as PubMed and SciELO, as well as public data from the Notifiable Diseases Information System (SINAN), official reports from the Ministry of Health and technical notes from the Amazonas Health Surveillance Foundation.

## RESULTS

### American Tegumentary Leishmaniasis (ATL) in the State of Amazonas

In Amazonas, ATL is primarily a zoonosis^7^, with its transmission cycle occurring between wild animals and phlebotomine sand flies. Humans generally acquire the disease accidentally when they enter the ecosystem of these animals and thus interfere with the sylvatic cycle^8^. *Leishmania (Viannia) guyanensis* is the most prevalent species in the cutaneous form, especially in the metropolitan region of Manaus, from which the vast majority of cutaneous leishmaniasis (CL) cases in the state of Amazonas originate^9^ ^-^ ^11^. Additionally, this species has also been recognized as an etiological agent of mucosal manifestations, along with *Leishmania (Viannia) braziliensis*, which is the main agent of this form of the disease in the Americas^6^ ^,^ ^12^. In Manaus, *Nyssomyia umbratilis* and *Nyssomyia anduzei* are considered to be the primary and secondary vectors, respectively, of *L. (V.) guyanensis* ^13^. 

### Temporal Evolution and Current Situation

The first records of leishmaniasis in the state of Amazonas were reported by Alfredo da Matta in 1918 (cited by Paes, 1998^14^) Subsequently, there was a marked surge in cases beginning in the late 1960s, particularly throughout the 1970s, a period marked by the consolidation of the Manaus Free Trade Zone and the intensification of human migratory movements driven, among other factors, by major floods in the main rivers of the Amazon basin^13^ ^,^ ^14^. This scenario favored the occupation of forested areas and increased contact between human populations and vectors, contributing to the spread of the disease. Thus, the expansion of leishmaniasis in the region is closely associated with deforestation, extractive activities and unplanned urbanization in forest frontier zones. In addition, the disease shows a seasonal pattern, with higher incidence rates during the rainy season^14^ ^-^ ^17^. 

Between 2001 and 2010, the state stood out for its high incidence of cases, with an annual average of 2,149 reports^11^. Among the factors contributing to the increasing number of cases, the large number of human settlements is noteworthy, as they are often established in highly vulnerable areas with poor sanitary conditions and close contact with natural environments where vectors and reservoirs are present, thereby facilitating disease transmission^18^. In addition, environmental factors such as changes in climate patterns have also contributed to the worsening of the situation, favoring the transmission cycle of leishmaniasis in the region^19^.

**FIGURE 2:**
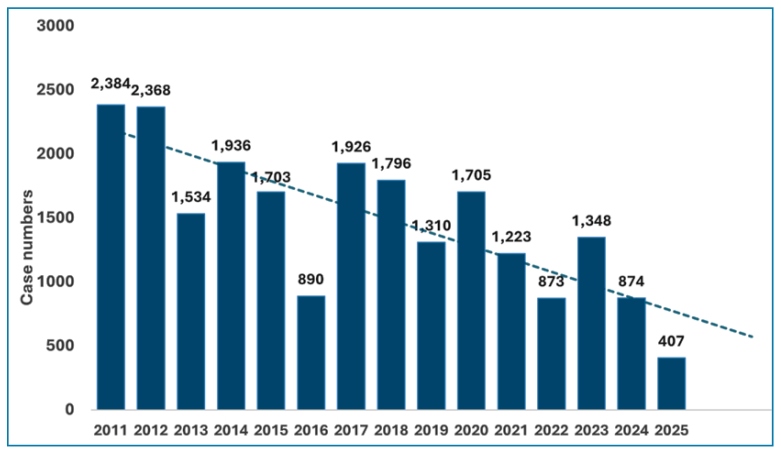
Distribution of cases of ATL in the state of Amazonas between 2011 and 2025. Data up to October 2025. **Source:** Sistema Nacional de Informações e Agravos (SINAN), 2011 - October 2025.

Between 2011 and 2025, a progressive reduction in leishmaniasis cases was observed in the state, with an annual average of 1,618 notifications, maintaining a downward trend (Figure 2). Specifically, the year 2016 was characterized by severe drought conditions, marked by an intense dry season and minimal rainfall, which likely had a direct impact on vector populations. Jiménez-Muñoz et al. (2016)^20^ highlighted that the 2015-2016 El Niño event caused unprecedented warming in the Amazon rainforest, with record-breaking temperatures and a broad geographical extent that significantly impacted evapotranspiration.

However, the manifestation of this event was not uniform across the basin. Specifically in 2016, the Amazon faced an extreme contrast scenario known as a "wet-dry dipole." While the eastern and northeastern portions experienced severe drought and record warming-increasing potential evapotranspiration-western Amazonia recorded atypical wetting with intense rainfall. This phenomenon, attributed to the location of the maximum warming in the Central Pacific Ocean, generated distinct climatic anomalies depending on the region of the state.

This climatic duality is fundamental to understanding why the reduction in the prevalence of American Tegumentary Leishmaniasis (ATL) in 2016 involves a complex interaction in transmission dynamics. Although warm and rainy periods favor the increase of sandflies^21^ ^,^ ^22^ phenomena such as El Niño impose variations that disrupt the enzootic cycle^19^. In 2016, both the excess rainfall in the west and the irregular patterns in the east may have acted as biological limiters, disrupting breeding microhabitats in the soil and reducing vector density. Concurrently, such anomalies influence the behavior of sylvatic reservoirs and modify human activity, reducing risk exposure in forested areas.

The influence of these climatic events on ATL incidence is corroborated by local studies, such as the one conducted in Manaus between 1990 and 2017^19^. This analysis demonstrated that the disease dynamics are directly influenced by El Niño-Southern Oscillation (ENSO) events, showing that during La Niña periods, the increase in rainfall starting in November precedes a rise in cases in the first quarter of the subsequent year.

**FIGURE 3:**
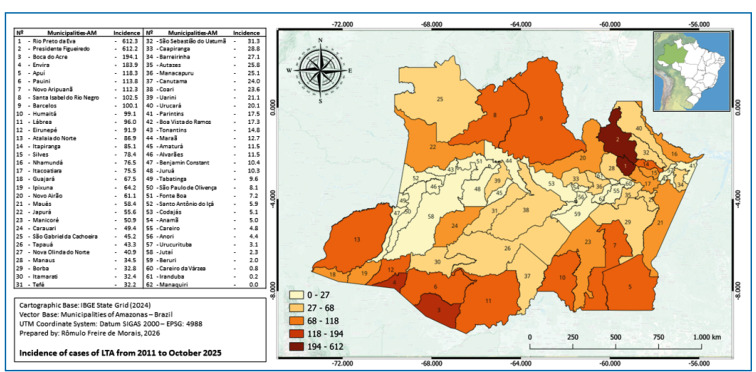
Map of ATL incidence by municipality in the state of Amazonas. Source: Sistema Nacional de Informações e Agravos (SINAN), 2011 - October 2025.

Additionally, there was an intensification of control measures related to population invasions and the consequent deforestation in the metropolitan region of Manaus, driven by initiatives from governmental agencies at both the state and federal levels, such as the Brazilian Institute of Environment and Renewable Natural Resources (IBAMA). However, despite the improvement, the highest concentration of cases still occurs in the metropolitan region of Manaus and in municipalities located along the main highways of the state, AM-010 and BR-174. In the state, Manaus continues to be the municipality with the largest number of cases-7,779, accounting for 39.6% of the total-ranking 29^th^ in incidence among municipalities. Presidente Figueiredo, Rio Preto da Eva, and Boca do Acre are, respectively, the municipalities with the highest incidence. This highlights the need for targeted surveillance and control actions in municipalities that are at a higher risk and also reflects the uneven distribution of the disease in the region (Figure 3). Among the most vulnerable population groups are rural workers, farmers, forestry workers and fishers, who frequently have direct contact with high-risk environments (Table 1). The age group with the highest prevalence is 21 to 30 years with 4,755 cases (24.2%), a stage of life characterized by economic activity and greater exposure to the sources of disease transmission, followed age group between 11 and 20 years old with 4,138 cases (21.1%).

**TABLE 1:** Occupation records - ATL cases in Amazonas from 2011 to 2022.

Occupation	Cases	%
Not recorded	12,989	66.11
Agricultural and forestry workers and fishers	4,666	23.75
Industrial goods and services production workers	809	4.12
Service workers and salespeople in shops and markets	408	2.08
Mid-level technicians	227	1.16
Science and arts professionals	205	1.04
Administrative services workers	127	0.65
Repair and maintenance services workers	85	0.43
Senior public officials, organization leaders and public interest representatives	71	0.36
Armed forces personnel, police officers and firefighters	61	0.31
Total	19,648	100

Source: Sistema Nacional de Informações e Agravos (SINAN), 2011 - 2022.

### Evolution of Scientific Knowledge in the Treatment and Control of ATL in the state of amazonas

The treatment of leishmaniasis in the Brazilian Amazon is standardized by the Brazilian Ministry of Health, and the control of case frequency depends on a combination of individual protection measures and public health actions. 

### Therapeutic options

The treatment of the different clinical forms of ATL should be initiated after diagnostic confirmation and is primarily based on the use of systemic drugs such as pentavalent antimonials, pentamidine, miltefosine and amphotericin B. 

Antimonials have been the first-line drugs for all forms of the disease in Brazil for more than 50 years and are provided free of charge by the Brazilian Unified Health System (SUS). They are commercially available as N-methylglucamine antimoniate (known as Glucantime®) and sodium stibogluconate (Pentostam®). The standard therapeutic regimen with pentavalent antimonials (10 to 20 mg/kg/day) for 20 days, administered intravenously or intramuscularly, is used for the treatment of localized and disseminated cutaneous forms. This procedure requires the availability of trained professionals and/or healthcare services for drug administration. 

In Amazonas, most patients come from rural and remote areas, and therefore completing the therapeutic regimen is often challenging, especially because, according to the Brazilian Ministry of Health, if complete healing does not occur within three months after the initial treatment, the regimen should be repeated for an additional 30 days. If therapeutic failure persists, one of the second-line drugs should be used^23^.

In this state, although meglumine antimoniate has traditionally been considered the first-line therapy for ATL, the Ministry of Health (MH) recommends pentamidine as the drug of choice for *Leishmania (Viannia) guyanensis*, at a dose of 4 mg/kg/day, administered in three doses on alternate days. However, its efficacy is still considered unsatisfactory, reaching approximately 58% in most cases^24^ ^-^ ^26^.

Considering the limited access to healthcare services among the populations most affected by ATL, the search for new therapeutic alternatives is of great importance. In Amazonas, several studies have been conducted, including *in vivo* and *in vitro* experimental research. Scientists at the National Institute for Amazonian Research have tested various substances to evaluate their potential use against *Leishmania* species. Among them, hesperidin has shown anti-*Leishmania* activity, although results are still preliminary^27^. There are also studies showing that an ointment containing a copper complex was effective in reducing lesion size in animals infected with *Leishmania (V.) guyanensis* and *Leishmania (V.) braziliensis*, suggesting that combination therapy with this formulation is promising for the treatment of these infections and its potential use in clinical practice is recommended^28^. 

### Clinical trials

Between 2011 and 2025 clinical trials were conducted in the Amazonas state, some of which demonstrated differences in cure rates, showing that the drug recommended by the Ministry of Health, pentamidine at a dose of 4 mg/kg body weight, has low efficacy, whereas higher doses of 7 mg/kg result in increased cure rates. 

**FIGURE 4:**
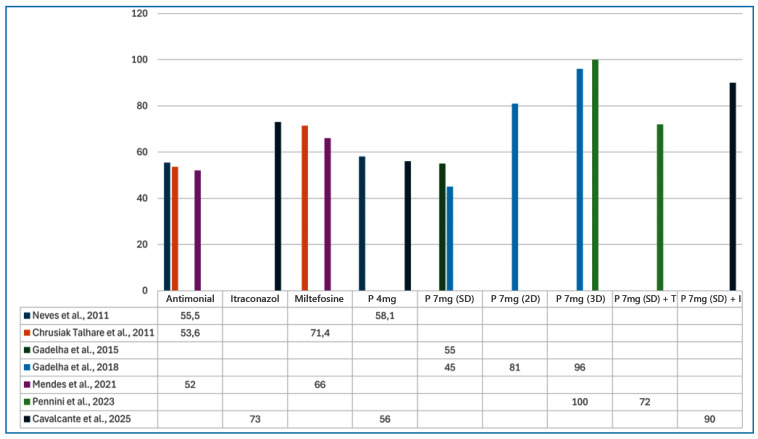
Description of cure rates observed in clinical trials conducted in the state of Amazonas between 2011 and 2025. **A:** Antimonial, **I:** Itraconazole, **M:** Miltefosine, **P:** Pentamidine, **T:** Tamoxifen, **SD:** Single dose, **2D:** Two doses, **3D:** Three doses.

For more than a decade, researchers from the Dr. Heitor Vieira Dourado Tropical Medicine Foundation (FMT-HVD) have conducted studies whose results clearly demonstrate the challenges in managing and treating leishmaniasis in this region (Figure 4) the clinical trials carried out in the state of Amazonas were as follows:

In 2011, Neves et al.^25^ reported that pentamidine and antimonials showed similar efficacy (58% and 55%, respectively) in the treatment of leishmaniasis caused by *L. (V.) guyanensis*. However, pentamidine is preferred over antimonials because treatment duration is shorter and it has fewer adverse effects. Nevertheless, both drugs-particularly pentamidine-require appropriate settings and qualified professionals for administration, a procedure that is often challenging in remote regions. 

In parallel to the study by Neves et al.^25^, Chrusciak-Talhari et al.^29^ published a study comparing the efficacy of a parenterally administered drug, an antimonial and an oral drug, miltefosine. The results showed six-month follow-up cure rates of 71.4% for miltefosine and 53.6% for the antimonial, highlighting the low cure rate with the antimonial and suggesting that miltefosine can be considered a viable treatment option. This perspective broadened the possibility of including an oral drug with better efficacy in the treatment of ATL in Amazonas. 

In 2015, Gadelha et al.^30^ published the results of a pilot study in which patients were treated with a single 7 mg/kg intramuscular dose of pentamidine diisothionate. Clinical cure was observed in 55% of the 20 patients enrolled in the study. 

In 2018, a new study was published by Gadelha et al.^31^. A total of 159 patients were evaluated, divided into three groups treated with 7 mg/kg intramuscular doses at seven-day intervals. The authors reported cure rates of 45% for the single-dose group, 81% for the two-dose group, and 96% for the three-dose group, demonstrating that pentamidine at 7 mg/kg administered in three weekly doses has the highest efficacy. 

In 2021, Mendes et al.^32^ compared the use of a placebo plus miltefosine (P + M), topical granulocyte-macrophage colony-stimulating factor (GM-CSF) plus miltefosine (G + M), and parenteral meglumine antimoniate (MA) in the treatment of 150 patients with leishmaniasis in Amazonas. The cure rates were 66% for the P + M group, 58% for the G + M group, and 52% for the MA group.

### Studies of potential leishmanicidal compounds:

Francesconi et al. (2018)^33^ evaluated the use of fluconazole at a dose of 450 mg/day for the treatment of cutaneous leishmaniasis caused by *Leishmania guyanensis*. Only one patient (5%) achieved cure within 30 days of treatment. Among the 19 cases of therapeutic failure (95%), 13 showed worsening of the ulcers and six progressed to lymphatic dissemination of the disease. Due to the high failure rate observed in the first phase of the study, the initially planned dose escalation was halted and the study was discontinued. The authors concluded that fluconazole is not effective for the treatment of leishmaniasis in the region. 

In 2023, Pennini et al.^34^ conducted an innovative study that evaluated the efficacy of the combination of tamoxifen and pentamidine in the treatment of leishmaniasis caused by *Leishmania (Viannia) guyanensis*. The intervention group received a single intramuscular dose of 7 mg/kg of pentamidine isethionate, followed by oral administration of tamoxifen citrate at 40 mg per day (20 mg every 12 hours) for 20 consecutive days. For comparison, the control group was treated with pentamidine isethionate, 7 mg/kg body weight, administered in three intramuscular doses at seven-day intervals. In the primary outcome, a cure rate of 72% was observed in the intervention group and 100% in the control group. It is noteworthy that this study resulted from a partnership between the Dr. Heitor Vieira Dourado Tropical Medicine Foundation (FMT-HVD) and the Alfredo da Matta Hospital Foundation (FUHAM), highlighting the joint effort to improve therapeutic alternatives in the region.

Cavalcante et al. (2025)^26^ conducted a study evaluating the use of itraconazole alone and in combination with a dose of pentamidine in the treatment of leishmaniasis cases caused by *L. (V.) guyanensis*. Patients treated exclusively with oral itraconazole (400 mg/day for 60 days) achieved a cure rate of 73%. Another group received a combination of a single intramuscular dose of pentamidine isethionate (7 mg/kg) and oral itraconazole (400 mg/day for 60 days), reaching a cure rate of 90%. A control group received three intramuscular doses of pentamidine (4 mg/kg once weekly), achieving a cure rate of 56%. Therefore, the itraconazole-pentamidine combination could be a therapeutic alternative for cutaneous leishmaniasis in the Amazon.

These findings underscore a proactive research effort to overcome the therapeutic challenges of leishmaniasis in the Amazon. The transition from ineffective treatments, such as fluconazole, to more robust regimens like the pentamidine-itraconazole combination, reflects a critical move toward optimizing care in a high-endemicity region. In a landscape where geographical barriers often limit access to healthcare, developing highly efficacious and shorter treatment protocols is essential. Such advancements not only improve clinical outcomes but also address the socio-economic vulnerability of populations who face significant obstacles in achieving therapeutic success.

### Mapping of Available Resources in Amazonas and Scientific Advances

Over the past twenty years, scientific advances in the study of leishmaniasis in the Amazon have been driven by a series of highly relevant research activities. The majority of funded projects are supported by the Amazonas State Research Foundation (FAPEAM), which also provides fellowships for researchers, including undergraduate, master’s, and doctoral students. Although not all projects receive direct funding, these fellowships constitute a significant financial incentive from the Foundation. Regarding other agencies, projects have been funded by the National Council for Scientific and Technological Development (CNPq), the Coordination for the Improvement of Higher Education Personnel (CAPES), and the Brazilian Innovation Agency ((FINEP). A smaller percentage of funding comes from other organizations, such as the Inter-American Development Bank (IDB), which funded the development of a mobile application to assist in the diagnosis of ATL.

Since 2003, FAPEAM has played a fundamental role in providing essential financial support for the development of research projects aimed at understanding the epidemiology, biology, treatment, and control of the disease in the region. Its creation and support fostered the training of human resources and promoted the generation of local and regional knowledge, essential factors for tackling leishmaniasis in the Amazonian context. By creating a more favorable environment for research and innovation, the creation of FAPEAM represented a milestone, facilitating the achievement of relevant scientific results and contributing significantly to strengthening disease control and prevention strategies in the Amazon.

Direct microscopic examination of lesion border scrapings has been considered the gold standard for the diagnosis of cutaneous leishmaniasis (CL); however, factors such as lesion duration and the technician’s level of expertise may influence diagnostic accuracy. Espir et al. (2016)^35^ evaluated the effectiveness of different diagnostic methods for CL in the state of Amazonas between 2010 and 2011, analyzing 38 suspected cases diagnosed using multiple techniques. The authors reported positivity rates of 71.0% (n = 27) for direct examination and 75.6% (n = 28) for culture; among culture-positive samples, 100% were positive by polymerase chain reaction (PCR), and 50% (n = 19) were identified as *Leishmania (Viannia) guyanensis*. The Montenegro skin test was positive in 77.0% of the evaluated cases, while serological testing for IgG and IgM antibodies demonstrated antibody presence in 100% of the performed assays. These findings indicate that the combined use of diagnostic methods is recommended to achieve more effective and earlier diagnosis, depending on the clinical or epidemiological question being addressed.

Diagnostic tests employing recombinant antigens derived from cases of American tegumentary leishmaniasis (ATL) in Brazil-19.5% of which originated from the state of Amazonas-have also been explored, as demonstrated in the study by Valencia-Potillo et al. (2024)^36^, which aimed to improve ATL diagnosis. In this study, the rLb6H-ELISA for IgG antibody detection was validated using 1,091 samples from patients with leishmaniasis and healthy controls, divided into four groups and residing in 19 endemic and non-endemic Brazilian states. The rLb6H-ELISA demonstrated a sensitivity of 98.6% and a specificity of 100.0% when evaluated against a reference panel consisting of 70 ATL patient samples and 70 healthy controls.

Studies on the identification of *Leishmania* species have been conducted primarily in collaboration with FUHAM, with spatial distribution mapping of the less frequent species^26^ ^,^ ^29^ ^,^ ^34^.

Regarding the etiological agents, research has identified, for the first time in Amazonas, species such as *L. (V.) shawi* and *L. (V.) lainsoni*, in addition to reinforcing the role of *L. (V.) naiffi* in the etiology of cases in Manaus and its surrounding regions^29^ ^,^ ^37^. 

Most suspected cases of mucosal leishmaniasis (ML) are primarily diagnosed based on clinical and epidemiological criteria, with histopathological examination being required as a complementary approach from which additional methods and techniques are employed to establish the etiological diagnosis. This is necessary because the detection of parasites in mucosal tissue is rare and, in some contexts, has even been used as a criterion to raise suspicion of HIV infection associated with ATL. 

Notably, Guerra et al. (2011)^12^ demonstrated that 65.2% of reported ML cases in the Amazon region are caused by *L. (V.) braziliensis*, while *L. (V.) guyanensis* accounts for 34.8%.

Among studies on socio-environmental aspects, an evaluation was conducted in the Purus region, including municipalities in Acre, assessing occupational exposure to the transmission cycle and the occurrence of cutaneous and mucosal forms of leishmaniasis. In this area, 20.8% of cases were of the mucosal type during the study period^38^. 

In the field of genetics, studies have demonstrated the influence of genetic markers on the susceptibility of the Amazonian population to American tegumentary leishmaniasis (ATL). *Leishmania (Viannia) guyanensis*, has been reported to exhibit greater resistance to antimonial derivatives compared with other species, such as *L. (V.) braziliensis* ^39^ ^-^ ^41^ *.* Consequently, pentamidine has been recommended as the first-line treatment in the region. However, in recent years, few studies have been conducted to reassess or challenge this assumption, particularly those evaluating the resistance profiles of circulating species. Nonetheless, several investigations have focused on the susceptibility of parasite populations by analyzing specific genetic markers associated with drug response.

Moreover, the expansion of telemedicine has proven very promising for the follow-up of cases in remote municipalities, as demonstrated by Pennini et al. (2025)^42^. This work also opens perspectives for new research avenues using artificial intelligence, as has recently been conducted in partnership with the Brazilian Israeli Beneficent Society -Albert Einstein Hospital, in the development of a tool to assist in the clinical diagnosis of ATL using smartphones.

A study conducted by the FMT-HVD Leishmaniasis group, in partnership with Amazonas State University (UEA) and Amazonas Health Surveillance Foundation - Dr. Rosemary Costa Pinto (FVS-RCP-AM), indicated that leishmaniasis vectors, although not yet colonizing households, are present in forested areas near human environments. Species such as *N. umbratilis*, the main vector of *Leishmania guyanensis*, were detected, increasing the risk of transmission. The study also detected parasite DNA in non-vector species, evidencing parasite circulation in these environments and reinforcing the need for continuous monitoring in the region^43^.

Researchers from the Leônidas and Maria Deane Institute of the Oswaldo Cruz Foundation in Manaus evaluated the microbiota of phlebotomine sand flies in two cities near Manaus, one with a high and the other with a low incidence of leishmaniasis. The authors identified specific bacteria in each phlebotomine population; notably, some species previously described in the literature as having parasiticidal properties were found exclusively in the municipality with lower case frequency. These findings highlight molecular and microbial peculiarities that may significantly explain the differences in leishmaniasis prevalence between the two regions^44^ ^,^ ^45^. 

### Control, Prevention and Public Polices Strategies

Regarding the control of leishmaniasis in Amazonas, this is a task that involves integrated surveillance, healthcare, and prevention actions, with a focus on interrupting the sylvatic transmission cycle, which includes multiple species of phlebotomine vectors^46^ and reservoirs^47^. Most cases originate in remote areas, where affected individuals were exposed to the disease transmission cycle during their occupational activities^12^ ^,^ ^26^ ^,^ ^32^ ^,^ ^42^. 

In the state of Amazonas, control actions have been carried out by FVS-RCP-AM in collaboration with municipal surveillance departments. In addition to case diagnosis and treatment, these actions have primarily focused on the continuous training of multidisciplinary teams, including community health workers-who play a key role in on-site case follow-up and in tracing patients in need of follow-up or those who have discontinued treatment-as well as laboratory technicians responsible for specimen collection and diagnostic procedures for suspected cases. Furthermore, training has been provided to healthcare professionals involved in patient care, particularly in primary healthcare units, including physicians, nurses, pharmacists, biochemists, and dentists.

With regard to vectors, limited control actions have been implemented, as outbreaks or epidemic situations have not occurred in recent years, with only sporadic cases being reported, sometimes affecting members of the same household. Consequently, vector-related activities have been largely restricted to research initiatives.

With regard to public policies, notable measures include the strengthening of training initiatives for healthcare professionals, combined with the decentralization of care to primary healthcare units. These units also provide health education to the population through educational talks for users, facilitating patients’ access to services closer to their areas of residence. Additionally, measures have been implemented to address unplanned land invasions on the outskirts of urban centers and to combat deforestation.

It is also noteworthy that, in addition to advances in treatment, the expansion of the diagnostic and treatment/follow-up network for leishmaniasis cases in primary healthcare units in the state capital, Manaus, was an important step. Previously, care was limited to only three facilities: FMT-HVD, FUHAM, and the Araújo Lima Outpatient Clinic.

Another important development was the publication of a technical note (TN) providing guidance on the treatment of leishmaniasis throughout the state of Amazonas. The first leishmaniasis TN, No. 027/2025/DVE/DIPRE/FVS-RCP, strengthened epidemiological and laboratory surveillance, treatment, prevention and control of leishmaniasis in the state. 

This TN addresses the strengthening of epidemiological and laboratory surveillance, treatment, prevention, and control of ATL in the state of Amazonas. It aims to guide health professionals regarding the laboratory diagnostic workflow, epidemiological surveillance actions, and available therapeutic approaches, with a view to ensuring appropriate clinical management and timely case notification^48^.

### Agenda For Priority Research on Leishmaniasis in the Coming Years

Despite advances in leishmaniasis control-particularly through service decentralization and the expansion of care within the Primary Health Care Network in Manaus and other municipalities-significant structural and operational challenges persist. Notably, insufficient training of health professionals and limitations in service infrastructure continue to hinder the implementation of second-line therapeutic regimens and alternative treatment approaches.

In this context, this article recommends prioritizing the following actions:

- Continuous training of health professionals, ranging from community health workers to personnel involved in decentralized, high-complexity diagnostic services.
- Development and validation of user-friendly educational materials and virtual platforms tailored to professionals working in remote and hard-to-reach areas.
- Strengthening comprehensive support for patient care, treatment, and long-term case monitoring.
- Incorporation of effective therapeutic regimens targeting circulating *Leishmania* species, particularly *Leishmania (Viannia) guyanensis*.
- Promotion of research projects integrated with the Unified Health System (SUS) and supported by the Amazonas State Research Support Foundation (FAPEAM).
- Expansion of molecular diagnostic methods, particularly to support the diagnosis of mucosal leishmaniasis using less invasive sampling approaches, such as those described by Cantanhêde et al. (2021^49^, and for accurate identification of *Leishmania* species circulating in the region.
- Support for technological innovation, including digital health tools, expansion of telemedicine-based diagnostics, and strengthening of the regional health care network.
- Strengthening connections between specialists and primary health care professionals through telemedicine, especially in remote municipalities, with regular continuing education and training sessions.
- Enhanced support for the diagnosis and follow-up of mucosal and cutaneous-mucosal leishmaniasis (ML/CML), ensuring access to specialist care (e.g., otolaryngologists) for diagnostic procedures such as biopsy and imaging, as well as the establishment of effective referral and counter-referral systems-particularly for cases originating in rural and interior areas of the state.
- Addressing the persistent shortage of qualified health professionals, which continues to compromise treatment delivery and case follow-up due to workforce discontinuity resulting from retirements and insufficient replacement, representing a critical gap that requires strategic intervention.

Collectively, these actions are expected to improve clinical management and strengthen public policies aimed at the effective control of leishmaniasis in the Amazon region, while accounting for regional specificities such as environmental determinants, therapeutic response, and host genetic susceptibility.

## CONCLUSIONS

This narrative review synthesizes the primary epidemiological, clinical, diagnostic, and therapeutic advances in American Tegumentary Leishmaniasis (ATL) within the state of Amazonas from 2011 to 2025. Although reported cases have steadily declined over the past decade, ATL remains a significant public health challenge. It persists notably in rural and recently deforested areas, disproportionately impacting young, economically active populations.

The findings underscore the predominant role of *Leishmania (Viannia) guyanensis* in regional epidemiology and highlight critical limitations of the current first-line therapy-pentamidine at 4 mg/kg-which is frequently associated with suboptimal cure rates. Conversely, studies conducted in Amazonas demonstrate the high efficacy of pentamidine administered at 7 mg/kg (three weekly doses), achieving cure rates exceeding 90%. Furthermore, combination therapies, such as pentamidine plus itraconazole, have shown promising results and greater feasibility for implementation within decentralized health services.

Given this evidence, a formal evaluation by regulatory bodies, such as the Ministry of Health, is warranted to consider incorporating these therapeutic alternatives for ATL caused by *L. (V.) guyanensis*. Such updates should be coupled with strengthened pharmacovigilance and operational research. Additionally, this review notes significant progress in diagnostic capacity, particularly through molecular techniques and minimally invasive approaches, alongside the expanding role of telemedicine in enhancing access to specialized care.

## Data Availability

Research data is available in the body of the article.
